# How does nature exposure make people healthier?: Evidence for the role of impulsivity and expanded space perception

**DOI:** 10.1371/journal.pone.0202246

**Published:** 2018-08-22

**Authors:** Meredith A. Repke, Meredith S. Berry, Lucian G. Conway, Alexander Metcalf, Reid M. Hensen, Conor Phelan

**Affiliations:** 1 Department of Psychology, University of Montana, Missoula, Montana, United States of America; 2 Behavioral Pharmacology Research Unit, Department of Psychiatry and Behavioral Sciences, Johns Hopkins University School of Medicine, Baltimore, Maryland, United States of America; 3 College of Forestry and Conservation, University of Montana, Missoula, Montana, United States of America; Hong Kong Baptist University, HONG KONG

## Abstract

Nature exposure has been linked to a plethora of health benefits, but the mechanism for this effect is not well understood. We conducted two studies to test a new model linking the health benefits of nature exposure to reduced impulsivity in decision-making (as measured by delay discounting) via psychologically expanding space perception. In study 1 we collected a nationwide U.S. sample (n = 609) to determine whether nature exposure was predictive of health outcomes and whether impulsive decision-making mediated the effect. Results indicated that Nature Accessibility and Nature Exposure From Home significantly predicted reduced scores on the Depression, Anxiety, Stress Scales (DASS) (*p* < .001, *p* = .03, respectively) and improved general health and wellbeing (*p* < .001, *p* < .01, respectively). Nature Accessibility also predicted reduced impulsive decision-making (*p* < .01), and Nature Accessibility showed significant indirect effects through impulsive decision-making on both the DASS (*p* = .02) and general health and wellbeing (*p* = .04). In Study 2, a lab-based paradigm found that nature exposure expanded space perception (*p* < .001), and while the indirect effect of nature exposure through space perception on impulsive decision-making did not meet conventional standards of significance (*p* < .10), the pattern was consistent with hypotheses. This combination of ecologically-valid and experimental methods offers promising support for an impulsivity-focused model explaining the nature-health relationship.

## Introduction

Human history evolved around an intimate connection to the natural environment (e.g., see [1,2]). This has changed dramatically over the last century. The recent shift to over half of the world’s population living in urban areas [3] together with advancements in technology (e.g., see [4,5]) has drastically reduced the amount of time many people spend in contact with nature. This separation of humans from nature may not be inconsequential. A growing body of research is dedicated to exploring how interactions with the natural environment affect human health and wellbeing [6]. To date, researchers have demonstrated that humans gain a plethora of health and wellbeing benefits from nature exposure. Here, we present two studies that test a new theory linking the health benefits of nature exposure to reduced impulsivity via psychologically expanding space perception.

### Nature exposure and human health

A wealth of evidence exists linking nature exposure to improved health outcomes. From reduced recovery time following surgery [7] to reduced Attention Deficit Hyperactivity Disorder symptomology in children (e.g., [8–12]), health improvements in patients with cancer (e.g., increased expression of anti cancer proteins [13]) and cardiovascular disease (e.g., reduction of hypertension [14,15]) and many other outcomes–the abundance of evidence for this effect is striking. While much of the research exploring the effects of nature exposure has focused on psychological effects (see e.g., [16–19]), there is also considerable research linking nature exposure to improved physiological health markers. For example, exposure to nature images has been linked to decreased oxyhemoglobin concentrations (which is believed to be associated with psychological calming) in the right prefrontal cortex [20], and favorable changes in heart rate variability [21]. Physiological effects have also been observed in studies testing the effects of various nature exposure therapies (for review of such studies conducted in Japan see [22]). In addition to effects associated with viewing nature scenes or participating in nature exposure therapy, research has also found evidence in favor of positive physiological effects of basic environmental exposure. For example, sunlight has been linked to vitamin D production, release of nitric oxide, production of beta-endorphin, and regulation of circadian rhythms [23].

Further, research has revealed nature not only improves a wide array of health and wellbeing outcomes, but that both experimentally manipulated increases in nature exposure (e.g. [15]) and nature exposure resulting from living and/or working within proximity to ample natural space (e.g., [24–26]) can have these impacts on health. Given this wealth of evidence linking nature exposure to human health and wellbeing, some researchers have shifted from asking whether nature exposure improves health, to asking how nature exposure improves health.

### Possible mechanisms

Before researchers had rigorously evaluated the effects of nature exposure, there was the “nature benefit assumption”[27]. This term captures the intuitive belief many held prior to recent decades–that nature exposure was generally good for humans. However, it was not until Wilson’s biophilia theory [28], which stated that humans have evolved to respond positively to unthreatening natural environments, that rigorous scientific inquiry began. Since the emergence of this theory, researchers have turned their attention to understanding the specific nature of these positive responses. As a result, a substantial body of research now confirms the basic tenet of the nature benefit assumption. With evidence that nature exposure predicts positive outcomes for humans, researchers have increasingly shifted to asking *how* it is that nature affects humans to bring about so many positive outcomes. As a result of this collective inquiry, evidence has been found in support of a variety of possible mechanisms underlying the positive effects of nature exposure. While many potential pathways have been studied (e.g. see [29,30] for reviews), much attention has been paid to restoration, social consequences, and physical activity as potential underlying mechanisms.

#### Restoration

Early research into the mediators of the nature exposure-health and wellness relationship revealed evidence that natural environments facilitate physiological, emotional and attention restoration [31]. From this line of research, two complementary theories have spawned: Stress Reduction Theory [32] and Attention Restoration Theory (ART [33]). ART posits that nature exposure encourages effortless brain function, which facilitates its recovery from fatigue. SRT focuses on the role of affect. In particular, SRT suggests that exposure to natural environments facilitates positively-toned emotional reactions, which in turn have a restorative effect.

Although suggesting somewhat different routes to restoration, both theories emphasize that nature is psychologically restorative. And, consistent with this, the restorative quality of nature has been identified as a mediator of the effects of nature exposure on a variety health and wellbeing outcomes, including emotional wellbeing (see e.g., [34]) and mental health (see e.g., [35]).

#### Social consequences

Others have considered social consequences as a potential mechanism underlying the health and wellbeing effects of nature exposure. This endeavor has revealed evidence suggesting nature exposure increases social cohesion [36], as defined by Forrest and Kerns [37] to refer to sense of community, shared norms and values, positive and friendly relationships, and feelings of being accepted and belonging. Similarly, research has also found nature exposure enhances social connections [38] and social contacts [39]. Further, there is evidence suggesting increased nature exposure predicts reduced feelings of loneliness and decreased frequency of feelings of inadequate social support, and that reduced loneliness and decreased feelings of inadequate social support in turn improve self-reported health, a number of health complaints, and mental health [35].

#### Physical activity

Finally, others have considered the possibility that nature encourages physical activity. Evidence has shown that nature increases engagement in physical activity [40,41], and that “green exercise” (physical activity that occurs while being directly exposed to nature) improves health and wellbeing outcomes [42]. Although there is evidence in its favor, in a comparison of these three potential mechanisms (restoration, social cohesion, and physical activity) conducted by de Vries and colleagues [36], support was stronger for restoration and social cohesion as mechanisms than physical activity.

The present investigation–while acknowledging that the mechanisms discussed above play an important role in understanding how nature improves health and wellbeing–seeks to consider the role of another potentially important variable not directly addressed by restoration, social cohesion or physical activity: impulsive decision-making. The role of decision-making in predicting health and wellbeing outcomes is extremely important. Lifestyle factors such as diet, exercise habits, sleep routines, and stress management are largely the result of decision-making processes, and are critical predictors of health and wellbeing. Therefore, the present study does not seek to undermine existing theories regarding the underlying mechanism(s) for the health and wellbeing benefits of nature exposure, but rather further explores an alternate, and potentially complementary emerging hypothesis: that nature exposure improves decision-making, and that decision-making in turn improves health and wellbeing outcomes. As decision-making related to health can often be conceptualized as the choice between a more impulsive option (e.g., eating unhealthy food for dinner because it is faster and easier to prepare) and a less impulsive option (e.g., spending more time and energy making a healthy dinner from scratch), the present study will focus specifically on impulsive decision-making.

Importantly, impulsive decision-making may provide additional insight into the drivers of the effects of nature exposure on health, beyond what has been learned from prior research related to underlying mechanisms. Most notably, research on impulsive decision-making and restoration-related process such as stress (e.g., [43,44]) and fatigue (e.g., [45,46]) has found mixed results, suggesting that there is value in considering impulsive decision-making as a distinct process from restoration. This is not surprising because the processes, while potentially overlapping, are conceptually distinct. For example, one may feel restored but still choose to eat unhealthy food–indeed, research on positive emotion more broadly suggests that it can sometimes make people more susceptible to immediate cues [47]. By focusing on impulsive decision-making in the present investigation, we seek to directly study the effects of nature on such decision-making processes.

#### Impulsivity and impulsive decision-making

Impulsive decision-making has been identified as one component of the broader construct of impulsivity–a construct that has been defined through multiple psychological lenses. As noted by Evenden [48], impulsivity has been defined as both a character trait related to quick decision-making [49], a set of actions leading to poor outcomes [48], or as a choice to prefer smaller immediate rewards over larger delayed rewards [50]. Given this diversity regarding how impulsivity ought to be defined, many have argued that impulsivity is actually comprised of multiple distinct factors (see e.g., [48,51]. Research by Broos and colleagues [51] into these potential factors found evidence for three distinct aspects of impulsivity: self-reported impulsivity, impulsive action, and impulsive choice.

Impulsive choice, also referred to as impulsive decision-making, is a behavioral aspect of impulsivity that is associated with the preference for smaller immediate rewards over larger delayed rewards. A common measure of impulsive decision-making is delay discounting, which can be defined as the decrease in value of an outcome or reward with the increase in delay until its receipt [52]. An impulsive choice within a delay-discounting paradigm can be operationalized as the choice of a smaller sooner (e.g., receiving $10 today) over larger later reward (e.g., receiving $50 in one month). Degree of delay discounting is predictive of a host of specific behaviors and health outcomes including smoking, obesity, alcohol use, exercise frequency, and expected longevity (see e.g., [53–56]). Importantly, impulsive decision-making in the form of precipitous delay discounting has been implicated as a partial cause of costly chronic illness and premature death in the US (e.g., lack of exercise/physical activity, poor diet, tobacco use, and excessive alcohol consumption; e.g., [57–64]). Researchers have speculated that delay discounting is the most important laboratory predictor of real world health-related behaviors currently available [65].

#### Nature exposure and impulsive decision-making

In addition to the abundance of research linking impulsive decision-making in delay discounting to health, Van der Wal, Schade, Krabbendam, and van Vugt [66], and Berry and colleagues [67,68] have also found evidence linking exposure to nature images to reduced impulsivity in delay discounting tasks. Taken together, this prior research connecting nature to impulsive decision-making and impulsive decision-making to health outcomes provides evidence for a model that impulsive decision-making mediates the nature–health relationship. However, despite this evidence connecting nature exposure to impulsive decision-making and impulsive decision-making to health in separate studies, no prior work has simultaneously tested the links between nature exposure, delay discounting and health in the same study. While prior research has suggested a link between nature exposure and increased self-discipline [69], research suggests self-discipline and impulsive decision-making should not be considered synonymous constructs (see e.g., [70,71]).

Understanding how nature might affect health via impulsive decision-making represents a potentially important and largely unexplored mechanism for determining nature’s beneficial effects on personal and societal-level health and wellbeing, and may offer insight into targeted nature interventions for broader public health benefits. Therefore, the present investigation explores the role of impulsive decision-making as measured by delay discounting to better understand a mechanism determining how nature improves health by potentially reducing unhealthy impulsive decision-making. Study 1 considers impulsive decision-making as a possible mechanism underlying the effect of nature exposure on health. Study 2 then considers whether an expansion of space perception might be the underlying mechanism driving nature’s effect on impulsive decision-making.

## Study 1

While prior research provides evidence that nature exposure reduces impulsive decision-making, and separately, that reduced impulsivity in decision-making leads to improved health outcomes, a formal evaluation of the nature→impulsive decision-making→health path has not been conducted. Therefore, Study 1 addresses this gap by evaluating the importance of impulsive decision-making in the nature–health relationship. To do so, we test the degree that nature’s relationship with health is indirectly carried through impulsivity using standard methods [72,73]. Using a sample of participants from across the United States, we hypothesized that: (1) increased nature exposure would predict improved health outcomes, (2) increased nature exposure would predict reduced impulsivity in decision-making, (3) reduced impulsive decision-making would predict improved health outcomes, and (4) there would be an indirect effect of nature on health *through* impulsive decision-making.

### Study 1 methods

#### Participants

Six hundred and nine participants were recruited through *Mechanical Turk*. Participation was limited to US residents aged 18 or over. Of the 609 participants, 60% were female. The mean age was 36.39 years (*SD* = 12.08). The University of Montana Institutional Review Board approved all procedures.

#### Procedure

Participants began by reading the informed consent. Following agreeing to participate in the study, participants completed all measures and responded to basic demographic questions. All experimental data collection was programmed via Qualtrics (Provo, Utah).

### Study 1 measures

#### Nature exposure

Participants responded to thirteen questions assessing the degree to which they are exposed to nature in their daily lives. A principal component analysis using a Varimax rotation with Kaiser Normalization was performed on all items assessing nature exposure. Components with eigenvalues greater than or equal to 1 were retained. Three measures of nature exposure were identified through this process and are referred to as Nature Exposure From Home, Nature Accessibility, and Presence of Blue Space. The first component (Nature Exposure from Home) contained items related to the nature visible from one’s home, access to a yard with green elements (e.g., grass, trees, etc.), and assessments of the degree that one’s neighborhood contains green elements (*n* = 6, α = .90). The second component (Nature Accessibility) contained items assessing the prevalence of parks or other pleasant natural features nearby, the amount of time one spends outdoors, and how safe one feels being outdoors in the area around where they live (*n* = 5, α = .73). Finally, the third component (Presence of Blue Space) contained one item assessing whether one had access to a yard with a blue element (e.g., pond, creek, etc.) and another item measuring how one’s neighborhood compared to areas in presence of blue elements (*n* = 2, α = .65). Items were developed by research team and are similar to methods used in prior research (e.g. [74,75]). See S1 Appendix for list of items.

#### Geospatial nature proximity

In addition to the self-report items, we also quantified the natural land cover surrounding respondents’ home addresses using remotely sensed data, aggregated at three nested scales. Respondents were asked to provide their home address in an optional response item (61% of participants reported some portion of an address, ranging from street address to zip code). Addresses provided were geocoded using ESRI ArcMap 10.3.1. Natural land cover in the vicinity of address locations was assessed using the National Land Cover Dataset (NLCD), a spatially explicit raster database with 30-m resolution depicting land cover and land cover change for the United States [76]. In this dataset, land cover is classified into 16 categories based on spectral signature and post-processing analysis, including: open water, perennial ice/snow, developed (4 categories), barren land, forest (3 categories), grassland/herbaceous, shrub/scrub, pasture/hay, cultivated crops, and wetlands (2 categories). We considered ‘natural land cover’ to be any 30-m raster cell classified as water, perennial ice/snow, grassland/herbaceous, shrub/scrub, pasture/hay, cultivated crops, and any forest or wetland. Percent natural land cover was calculated as the number of natural land cover cells divided by total cells within three nested spatial scales: immediate vicinity, neighborhood, and broader locality. Immediate vicinity was operationalized as any cell within 90m of respondents’ home address (participants who only provided a zip code were excluded from this level of analysis). Neighborhood and broader locality were operationalized as the Census block and County intersected by respondent’s home address, respectively.

Six variables resulted from this process, which we will refer to as: (1) Immediate Vicinity Green, (2) Neighborhood Green, (3) Broader Locality Green, (4) Immediate Vicinity Blue, (5) Neighborhood Blue, and (6) Broader Locality Blue. The ‘green’ items were all positively correlated and therefore combined into a summary variable, Geospatial Green Proximity (α = .72). The ‘blue’ items were not sufficiently correlated with one another, and therefore considered as separate variables for analyses.

#### Health measures

Participants completed two health measures: (1) The Depression Anxiety Stress Scales, or DASS [77] (DASS scores were reverse-coded during analysis, resulting in higher scores reflecting increased mental health), and (2) items designed to measure general health and wellbeing (derived from prior research [78]; α = .88).

#### Impulsive decision-making measure

To measure degree of impulsive decision-making, participants completed a delay-discounting task using hypothetical monetary outcomes. Participants responded to a series of questions asking “Would you rather have [amount of money] now, or [amount of money] in [delay]?” (see [79] for a detailed description of the descending fixed order delay discounting task). Participants chose either the immediate or delayed option. Immediate amounts were presented in a fixed descending order ($100, $99, $95, $90, $85, $80, $70, $60, $50, $40, $30, $20, $15, $10, $5, and $1). The delayed amount was constant at $100. All aforementioned values were presented at each delay (delays included 1 day, 1 month, 1 year, and 5 years).

Impulsive decision-making was quantified using Area Under the Curve (AUC; [80]). AUC is derived from normalizing the indifference points in the fixed sequence procedure, which are defined as the last immediate amount chosen at each delay, as well as normalizing the delays (see [80]). AUC scores range from 0 to 1, with higher numbers representing less impulsivity in decision-making (i.e., more willingness to wait for delayed, but larger outcomes), and lower numbers representing more impulsive decision-making.

#### Covariates

Participants were also asked to report their highest level of education achieved and household income as indicators of socioeconomic status.

### Study 1 results

#### Nature exposure on health

A series of simple linear regression analyses were initially used to test for an association between nature exposure and health. In all cases, to test for the additive impact of the variable above and beyond demographic factors, we further performed hierarchical regression where education and income were entered in Block 1 and the predictor of interest was entered in Block 2.

First, Nature Accessibility was significantly associated with improved mental health (β = .24, *t*[549] = 5.84 *p* < .001) and general health and wellbeing (β = .26, *t*[560] = 6.24, *p* < .001). Each of these effects showed the unique predictive value of Nature Accessibility when education and income were entered in Block 1 in hierarchical regression (*R*^2^ change *p*’s < .001). Similarly, Nature Exposure From Home was found to be significantly associated with improved mental health (β = .10, *t*[547] = 2.30, *p* = .02) and general health and wellbeing (β = .12, *t*[557] = 2.80, *p* < .01). Each of these effects showed the unique predictive value of Nature Exposure from Home when education was entered as a covariate in Block 1 in hierarchical regression (*R*^*2*^ change *p*’s < .05), while similar analysis using income as a covariate revealed non-significant added effects of Nature Exposure from Home (*R*^*2*^ change *p* = .15, *R*^*2*^ change *p* = .13, respectively). Finally, Presence of Blue Space (i.e., greater blue space) was associated with a marginally lower mental health scores, although this relation was not statistically significant (β = -.08, *t*[564] = -1.90, *p* = .06). Presence of Blue Space was not found to be associated with general health (β = .02, *t*[574] = .46, *p* = .64). See Table 1.

**Table 1:** Standardized effects of nature on health and impulsive decision-making via simple linear regressions.

		DASS	General Health & Wellbeing	Impulsive Decision-Making
Nature Exposure				
	Nature Accessibility	.24***	.26***	.11**
	Nature Exposure From Home	.10*	.12**	.02
	Presence of Blue Space	-.08	.02	-.03
Geospatial Nature Proximity				
	Geospatial Green Proximity	.01	-.03	-.01
	Immediate Vicinity–Blue	.04	-.02	-.04
	Neighborhood—Blue	.05	-.01	-.06
	Broader Locality—Blue	-.04	.00	-.04

*p < .05.

**p < .01.

***p < .001.

Impulsive Decision-Making measured by AUC (0–1). Higher values indicate less impulsive decision-making, and lower values indicate more impulsive decision-making (see text for details).

#### Nature exposure on impulsive decision-making

Consistent with expectations, Nature Accessibility was significantly associated with reduced impulsivity in decision-making (β = .11, *t*[587] = 2.63, *p* < .01). The effect of Nature Accessibility when education and income were entered in Block 1 in hierarchical regression was not significant, though in the expected direction (*R*^2^ change *p* = .06). Neither Nature Exposure From Home or Presence of Blue Space were significantly associated with impulsivity in decision-making (β = .02, t[584] = .36, *p* = .72; β = -.03, *t*[603] = -.70, *p* = .48).

#### Geospatial nature proximity

A series of simple linear regression analyses were used to test for an association between geospatial nature proximity and health. Inconsistent with expectations, none of the measures of geospatial nature proximity were associated with mental health or general health and wellbeing: Geospatial Green Proximity (β = .01, *t*[352] = .26, *p* = .79; β = -.03, *t*[356] = -.57, *p* = .57), Immediate Vicinity–Blue (β = .04, *t*[215] = .54, *p* = .59; β = -.02, *t*[213] = -.34, *p* = .73), Neighborhood–Blue (β = .05, *t*[352] = .90, *p* = .37; β = -.01, *t*[356] = -.12, *p* = .90), and Broader Locality–Blue (β = -.04, *t*[352] = -.79, *p* = .43; β = .00, *t*[356] = -.02, *p* = .99). Similarly, none of the measures of geospatial nature proximity were associated with impulsivity in decision-making: Geospatial Green Proximity (β = -.01, *t*[370] = -.14, *p* = .89), Immediate Vicinity–Blue (β = -.04, *t*[370] = -.61, *p* = .54), Neighborhood–Blue (β = -.06, *t*[370] = -1.09, *p* = .27), and Broader Locality–Blue (β = -.04, *t*[370] = -.75, *p* = .46). We return to the implications of these findings in the discussion. See Table A in S1 Appendix for correlations for all Study 1 variables.

#### Indirect effect nature exposure on health via impulsivity

A Sobel test [81] was conducted to determine whether there was an indirect effect of Nature Accessibility on health via impulsive decision-making. Sobel tests are used to test the significance of indirect effects, and in this case examined whether there was an indirect effect of Nature Accessibility on health via impulsive decision-making. Findings were consistent with expectations, as results revealed a significant indirect effect for both measures of health (mental health: *z* = 2.29, *p* = .02; general health and wellbeing: *z* = 2.02, *p* = .04). See Fig A in S1 Appendix for graphs.

### Study 1 discussion

Results from Study 1 include two key findings. First, the results demonstrate that greater nature exposure in the form of nature visible from home and the accessibility of nature in one’s area is associated with health benefits (and that this effect generally occurs above and beyond income and education level, as indicated by hierarchical regressions accounting for those demographic factors). And second, that part of the effect of nature accessibility on health is accounted for via indirect effects through impulsive decision-making. Specifically, these results indicate that a greater degree of nature accessibility is associated with less impulsivity in decision-making, and that less impulsivity in decision-making in turn is linked to improved health. These findings both replicate prior research related to the health benefits of living in proximity to nature, as well as extend our scientific understanding of the relationship between nature and health by providing promising evidence that impulsive decision-making may act as an underlying mechanism of the relationship for certain aspects of the nature–health relationship.

This result is especially impressive given the psychological distance between the impulsive decision-making measurement and our measurements of nature and health. The impulsive decision-making measurement pertained to hypothetical monetary amounts and thus was not directly related to either nature or health. It is in fact compelling support for our guiding theory that persons who report merely *subjectively experiencing more access to nature* show reduced impulsive choice on a measurement not related to nature. Given the ecological validity of measuring nature exposure in this way, these results suggest a fairly pervasive link between the degree of nature accessibility in the area around where one lives and reduced impulsive decision-making and improved health outcomes.

Finally, while the geospatial nature proximity measures did not produce results consistent with expectations, this was a relatively imprecise measure of nature exposure compared to the self-report items. Where the self-report items directly measured participants’ own experiences of nature in their daily lives, the geospatial analysis estimated this experience using low-resolution land cover data based on the area where one lives. Indeed recent research by Singh, Madden, Gray, and Meentemeyer [82] highlights an important shortcoming of using the NLCD for research on developed areas. However, regardless of the quality of the measures associated with the NLCD, these results did not directly support expectations. Some prior research using mapping technology has also failed to find an association between access to green space and wellbeing (see e.g., [83]). Future research should further explore differences between self-reported access to nature and the use of geospatial mapping technology to ascertain rates of nature exposure. While evidence suggests some geospatial mapping technologies may be better than others [82] at accurately measuring nature exposure in some areas (at least in the US), it is also possible that what matters more to outcomes such as wellbeing is one’s perception of nature exposure (which would be captured more accurately via self-report methods). Overall, more research is needed on this topic.

## Study 2

### Background

In Study 1, we found evidence for an indirect effect of nature exposure on health via impulsive decision-making. The relationship between impulsive decision-making and health is well-established in prior research; but less is known about nature’s impulsivity-reducing effect. Therefore, Study 2 focused on *the mechanism driving how* nature might influence impulsive decision-making. Prior research has found that time perception may not only be important in understanding changes in impulsive decision-making, but also that it may be influenced by exposure to natural environments [68]. However, despite these connections it has yet to be tested as a possible mechanism of the nature exposure–impulsive decision-making relationship. Further, time perception is one of many possible sensory experiences that might relate to both impulsivity and health. To help fill in these gaps, Study 2 tested (a) whether time perception might help explain changes in impulsive decision-making, and (b) whether another related sensory construct–space perception–might help explain changes in impulsive decision-making.

#### Time, impulsive decision-making, and nature

Given the ostensible importance of time in understanding impulsive decision-making, prior research has considered how various aspects of time might relate to impulsive decision-making. Evidence has been found that modification of temporal attention to be presently biased [84], episodic future thinking [85], and expanded time perception (e.g., [68,86]) all reduce impulsive decision-making. Of particular interest to the present investigation is the finding related to expanded time perception, as nature exposure has also been found to expand perception of time [68,87].

#### Space expansion: The overlap of time and space

Although research ties nature exposure to expansion of time, there is little research on the link between nature and perceptual expansion at a broader level. Yet it may be that fundamental characteristics of nature contribute to natural environments eliciting expansiveness in ways that go beyond time perception. For example, natural environments may be more likely on average to prime a sense of vast scale than non-natural environments and as such, contribute to perceptual expansion. To the degree that this is true, it may be that perceptual expansion might matter in more general ways. Therefore, Study 2 also considered a second variable related to perceptual expansion; the perception of space.

Given their common conceptual overlap, it is unsurprising that time perception and space perception are linked. Not only have studies linked perceptions of time and space under a variety of conditions, but a look at everyday language also suggests these constructs do not operate independent from one another. Many studies have considered the psychological linkage of space and time (for a review of this literature and a discussion of the neurological overlap of perceptions of space and perceptions of time, see [88]). In sum, there is an abundance of evidence that suggests human perception of space and perception of time are overlapping constructs (see e.g., [89–93]).

Further indicators of the association between space and time can be found in the linguistic overlap of terms describing time and space frequently used in everyday conversation. For instance, is it is common to hear time described using spatial terms or in an interchangeable way (e.g. ‘the end of the term is approaching’ or ‘short meeting’). This use of spatial language to refer to time is due to a richer perceptual experience humans have for matters of space. Many believe the reason for this is an intimate linkage of spatial and temporal representations in the mind (see e.g., [94–101]).

Given the psychological overlap of space and time, and the evidence for time perception’s role in predicting impulsive decision-making, it follows that space might provide useful insight into impulsive decision-making behavior. Further, the richer reasoning capabilities humans have for matters of space over time [102], and the tendency to conceptualize time in terms of space [103] offers reason to believe space may actually be a *more* efficacious variable than time for understanding impulsive decision-making. Despite these connections, however, investigations to date have not included inquiries into the possible predictive power of space perception in relation to impulsive decision-making.

Study 2 aimed to fill this gap by considering whether changes in either perception of time or perception of space were predictive of changes in impulsive decision-making, and whether there was a significant indirect effect of nature exposure on impulsive decision-making via either of these constructs.

#### Study 2 hypotheses

Study 2 considered whether exposure to nature might have a similar expanding effect on perceptions of space (as has been found with time), and whether there is evidence for an indirect effect of nature on impulsive decision-making via perception of time and/or perception of space. Specifically, this study used a nature exposure manipulation and delay discounting outcome measure to test: (1) the effect of nature exposure on time perception and space perception, (2) the effect of time perception and space perception on impulsive decision-making, and (3) time perception and space perception as possible mechanisms of the nature–impulsive decision-making relationship. We hypothesized that: (1) nature exposure would expand perceptions of time and space, (2) as time and space are perceived to be more expansive, impulsive decision-making would be reduced, and (3) the nature exposure-impulsive decision making relationship would be partially carried *through* both time perception and space perception.

### Study 2 methods

#### Participants

Sixty-six undergraduate students were recruited from undergraduate psychology courses. Participants provided written informed consent and received course credit for their participation. The University of Montana Institutional Review Board approved all experimental procedures.

#### Setting and apparatus

Participants completed the study alone in a small, quiet room. Four testing rooms were used. Each of the rooms was identical in size (115” x 64”) and layout, and each room contained two computers, two chairs, and a desk. Participants were asked to leave their personal belongings in a separate space to ensure engagement with the study and reduce distractions. Participants sat in front of a computer viewing images, reading prompts, and responding to questions that utilized E-Prime 2.0 and Qualtrics software.

#### Stimuli

Stimuli for the present study included images of nature scenes (e.g. lakes, forests, mountains) and images of built scenes (e.g., buildings, cities, roads). The built images served as a control condition for comparison to the nature scenes. These images have been used in previous investigations of attention restoration and delay discounting behavior (e.g. [66,67,104]).

#### Procedure

All experimental procedures were identical across natural and built conditions with the exception of the condition-specific photographs viewed. Participants were randomly assigned to either the nature condition or built condition. In each condition, participants viewed a series of images (each image was displayed for 10 seconds), based on their assigned condition both before and during the delay-discounting task (described in detail below). Prior to beginning the delay discounting task participants viewed 25 randomly presented condition-specific photographs. Between each delay block of the delay-discounting task, participants viewed 5 randomly presented condition-specific photographs selected from the original 25. Following completion of the delay-discounting task, participants responded to six questions related to perception of space and time, as well as demographic items.

#### Delay discounting task

To measure degree of impulsive decision-making, participants completed a delay-discounting task involving the choice between a fewer number of days of improved air quality now, versus a greater number of days of improved air quality after a range of delays (see [105] for a detailed description of the task). Participants used the computer mouse to select either a smaller air quality improvement immediately, or a larger air quality improvement after a delay. The immediate amount increased or decreased based on the participant’s response as previously described (see [79,106] for detailed descriptions of the titration procedure). Delays tested were 1 day, 1 week, 1 month, 6 months, 1 year, 5 years, and 25 years in that order. Impulsive decision-making was quantified using the same Area Under the Curve (AUC; [80]) analysis described in Study 1.

#### Perception of space

Participants were asked three questions designed by the research team to assess their perception of space. For the first space perception item, participants were asked to picture themselves immersed in the images they saw on the screen during the study and rate how the space around them felt (on a 1–10 scale [1 –constricted, 10 –expanded]). For the second space perception item, participants were asked to close their eyes for a couple moments and then rate how the space around them felt (on a 1–10 scale [1 –constricted, 10 –expanded]). Results from these two self-report space perception items were significantly positively correlated and were consequently combined into a two-item summary scale (α = .46) for analysis.

Lastly, an exploratory item was developed with the goal of gaining insight into participant’s perception of space without asking about it directly. This third space perception item asked participants to use a blank piece of paper and pen (identical paper and pen were provided to each participant by the researcher) to draw a ‘medium-sized circle’ from which the circle area was then calculated.

#### Perception of time

Three questions designed by the research team (and similar to methods used in prior research [68,87]) were used to assess participant perception of time: (1) How quickly has time seemed to pass since you first arrived and signed the informed consent? (response scale: 1 –time moved really slowly to 10 –time moved really fast), (2) How many minutes would you estimate have elapsed since you signed the informed consent?, and (3) Consider how time feels at this moment (response scale: 1 –time feels constricted to 10 –time feels expanded).

Responses to the two Likert-scale items were positively correlated, while the minute estimate of elapsed time was negatively correlated with both Likert-scale items. Due to the correlation between the items, they were combined into a three-item summary scale (α = .58) for analysis (the minute estimate time perception item was reverse coded prior to combination).

### Study 2 results

Of the 66 participants, 57% were female. The mean age was 23.62 years (SD = 7.79). Chi square (sex, race) and a *t* test (age) comparing demographics across natural and built conditions indicated no significant differences across groups for these variables (sex, *p* = .451; race, *p* = 1.00; age, *p* = .63).

#### Main effect of nature exposure on perception of space

Consistent with expectations, an independent samples *t*-test revealed a significant main effect of nature exposure on space perception, such that those exposed to nature images were more likely to report that the space around them felt expanded (*M* = 6.19, *SD* = 2.09) than those who viewed images of built scenes (*M* = 4.27, *SD* = 1.76), *t*(64) = 3.99, *p* < .001, *d* = 0.99. Also, given the exploratory nature of the circle-area item and its lack of significant correlation to other space items it was not included in the summary variable. However, the exclusion of this item does not change the pattern of results presented here: Testing a summary variable including the circle-area item also results in a statistically significant effect of condition on perception of space, *t*(64) = 2.33, *p* = .023, *d* = 0.57. See Table B in S1 Appendix for correlations between all space items.

#### Main effect of space perception on impulsive decision-making

A simple linear regression analysis was conducted to determine if space perception significantly predicted impulsive decision-making. Consistent with expectations, expanded space perception significantly predicted less impulsivity in decision-making, β = .31, *t*(1,63) = 2.57, *p* = .01.

#### Main effect of nature exposure on perception of time

An independent samples *t*-test revealed the main effect of condition on the time perception summary variable was not statistically significant, though it was in the expected direction. Those exposed to nature were somewhat more likely to report that time felt as though it was moving fast/expanded and that fewer minutes had elapsed than those exposed to built images, *t*(63) = 1.70, *p* = .09, *d* = 0.42.

#### Main effect of time perception on impulsive decision-making

A simple linear regression analysis was conducted to determine if time perception predicted changes in impulsive decision-making. Results approached statistical significance (β = .19, *t*[62] = 1.51, *p* = .14), such that increasingly expanded/lengthened time perception predicted reduced impulsive decision-making.

#### Analysis of the indirect effect of nature on impulsive decision-making through space perception

Given analysis of time perception did not reveal effects achieving conventional levels of statistical significance, only space perception was considered as a possible mechanism of the nature–impulsive decision-making relationship. Prior to conducting indirect effect analysis, an independent samples *t*-test was used to first establish the presence of a main effect of nature exposure on impulsive decision-making as measured by AUC (results of the discounting portion of this investigation are reported separately in Berry, Repke, & Conway [107]).

Finally, a Sobel test [81] was conducted to determine whether there was an indirect effect of nature on impulsive decision-making via space perception. Findings were somewhat consistent with expectations, though a significant indirect effect was not found, *z* = -1.66, *p* < .10. See Fig C in S1 Appendix for graph.

#### Perception of space—Perception of time relationship

While no hypothesis was made regarding the nature of the relationship of the perception of space and perception of time outcome measures, a correlation analysis was conducted for completeness. Summary variables of time and space perception were not found to be significantly correlated, *r* = .14, *p* = .27. Furthermore, additional analysis considering only items using the same response scale of ‘constricted’ to ‘expanded’ across the two domains were not found to be significantly correlated.

### Study 2 discussion

Overall, these results offer support for the framework under consideration: Nature exposure significantly predicted expanded space perception, and expanded space perception significantly predicted decreased impulsive decision-making. Support of the indirect effect of the nature on impulsive decision-making relationship via space perception was generally consistent with hypotheses, but somewhat weaker. These findings lend support to the broader theory underlying this investigation. They further suggest that humans’ superior perceptual abilities for spatial over temporal matters combined with the psychological relatedness of these constructs makes space perception an important variable in understanding and predicting impulsive decision-making.

## General discussion

The present studies are the first to offer evidence for the tenability of a model of nature’s health-boosting effects that focuses on impulsivity and perceptual expansion. Several important findings emerged. First, Study 1 provides evidence that the health benefits linked to nature exposure are partially explained by a reduction in impulsive decision-making. Second, Study 2 demonstrated that the effects of exposure to nature on decreased impulsive decision-making might be partially explained by expanded space perception. Taken together, these results specifically suggest nature exposure might expand space perception, which in turn may reduce impulsive decision-making, and that this reduction in impulsive decision-making might improve health outcomes.

The results of this study extend previous research by linking the indirect effects of nature exposure on health via impulsive decision-making and of nature exposure on impulsive decision-making via expanded space perception. These results may have important implications for our understanding of the effects of nature exposure on health outcomes by suggesting that with increased exposure to nature, impulsive decision-making, which might lead to stress, anxiety, depression, and reductions in overall wellbeing, might be reduced.

Second, these results are also the first to provide support for the involvement of space perception in the nature exposure–impulsive decision-making relationship presented in Study 2. Based on prior research, we considered time expansion to be an important variable in understanding the relationship between nature exposure and impulsive decision-making [68]. From there, we constructed a theory that since prior research in other contexts had linked the concepts of time and space, that space perception may be an important predictor of impulsive decision-making above and beyond time perception. This hypothesis was influenced by the idea that humans are more easily able to perceive matters related to space rather than time [108], so therefore we theorized that nature exposure was prompting a psychological expansion that was not isolated to time (and for which space perception might actually more acutely register).

The data from Study 2 suggest that we must qualify some of the assumptions on which this model was based. For instance, it is possible that exposure to nature influences perception of space differentially than perception of time such that even if nature exposure led to expanded perceptions of space and time, the reasons for these effects should not be considered equal, nor should the effects themselves be considered interchangeable (from a theoretical perspective). While this would not lessen the importance of the present findings, it would challenge the theoretical grounding presented here based on the overlap between space and time perception. Future research should be conducted to consider how perception of space and perception of time may or may not be related in their interactions with nature exposure and impulsive decision-making. Similarly, additional research should also test other assumptions such as potential other means for accessing psychological expansion beyond perception of space and time.

### Limitations

As with all studies, limitations exist. While the present investigation focused specifically on a mechanism (impulsive decision-making) potentially underlying how nature exposure might influence health via changes to health behaviors, health behaviors were not directly measured in this investigation. Further research testing the merits of the model presented here should include assessment of health behaviors rather than only self-reported health outcomes. Additionally, both of these studies were conducted on U.S. samples. Given that socioeconomic status has been linked to both health and impulsive decision-making, findings presented here should be considered within the context of a population that is more educated and wealthy, on average, than most others.

Further, the present studies were based on a causal mediational model, yet Study 1 employed a non-experimental design that does not allow for direct causal tests. The obvious drawback of this decision is the disconnect between the causal nature of the theoretical model and the non-causal nature of the study design. Indeed, it is important at a larger level to distinguish between the theoretical model–which is based on a mediational approach that assumes causality–and the statistical tests themselves. No statistical test of mediation can directly test the causal path assumed in a mediational model [109], and thus as with any such mediational model, caution is warranted. However, there are also benefits to the dual approach used here across Studies 1 and 2, which had complementary strengths and weaknesses. The non-experimental design of Study 1 allowed for a more ecologically-valid test of the effects of nature exposure–by looking at how natural environments in real-world contexts are actually related to health–compared to an experimentally manipulated environment. Further, in Study 1 we found an association between nature exposure and impulsive decision-making, which we replicated using an experimental design in Study 2. Indeed, the effects of Nature Exposure in Study 2 are more easily interpreted as causal, due to the randomized nature of the experimental design. Through the combined use of these different methods, we have demonstrated that this effect is likely to be both ecologically-valid and consistent with our hypothesized casual theory. But of course, more research is necessary to more completely validate the causal nature of this design; at this point, it is consistent with our causal mediational model, but it is possible that other possible explanations might yet also account for the found effects.

Finally, the present results were based on a model suggesting the effects of nature exposure on health are serially mediated through perceptual expansion and impulsive decision-making. Despite the evidence generally in favor of this model with regards to space perception, this entire model itself was not directly tested in the same sample. Future research assessing the role of impulsive decision-making and space perception as mechanisms of the nature exposure–health relationship should include a test of all of these constructs in a single design. The evidence presented here indeed suggests that impulsive decision-making acts as an underlying mechanism of the nature exposure–health relationship, and that space perception might act as an underlying mechanism of the nature exposure–impulsive decision-making relationship. Yet from these studies, we are not able to draw firm conclusions on how impulsive decision-making and space perception may interact with one another to predict health outcomes. Thus, these studies provide important evidence for many of the pieces of an emerging model, and future research would do well to test these pieces simultaneously.

### Conclusion

Prior research has linked a vast array of health effects to nature exposure. The present investigation sought to understand more about how nature might influence so many health outcomes by considering possible meditators underlying these effects. Using a combination of experimental and non-experimental methods, the studies presented here not only consider new mediators of these effects, but also lend further credence to the hypothesis that nature exposure plays a critical role in human health and wellbeing.

Future research on the health benefits of nature exposure and the underlying mechanism(s) stands to be theoretically as well as substantively important. As the evidence for the benefits of nature exposure in daily life mounts, additional opportunities for application are revealed in many fields including urban planning (e.g. [110]) and architecture/design both at home and in the workplace (e.g. [111]). These findings not only add to the abundance of evidence for this effect, but also demonstrate the diverse applications to many aspects of human life.

Finally, nature exposure may not only have important consequences for how humans treat themselves, but also how they treat nature (e.g. [112]). As our species is increasingly threatened by both anthropogenic climate change and epidemic levels of declining wellness, it is more important than ever to identify opportunities that ameliorate the effects of each as efficiently and effectively as possible. If simple nature exposure can simultaneously improve how humans choose to sustain their own health and wellbeing along with the health and wellbeing of the natural environment, it represents a unique and exceedingly important opportunity for researchers and society alike.

## Supporting information

S1 Appendix Supplementary information. (DOCX)
