# Supplementary material for: How does nature exposure make people healthier?: Evidence for the role of impulsivity and expanded space perception
Source: PLoS One. 2018 Aug 22;13(8):e0202246. doi: 10.1371/journal.pone.0202246 (PMC6104990; doi:10.1371/journal.pone.0202246)

General Health and Wellbeing items:

In general, how would you rate your health?

(1 = very poor, 7 = excellent)

How do you think your health compares to other people your age?

(1 = much worse, 7 = much better)

In general, I feel happy and/or content most of the time.

(1 = strongly disagree, 7 = strongly agree)

In general, I take good care of myself.

(1 = strongly disagree, 7 = strongly agree)

For the most part, I feel physically and mentally healthy.

(1 = strongly disagree, 7 = strongly agree)

Nature Exposure items:

From the main living space in your home, is the view outside the window(s) mostly of concrete, buildings, etc. or mostly of natural elements like trees, grass, etc?

(1 = mostly concrete, buildings, etc, 7 = mostly trees, grass, etc)

From your bedroom in your home, is the view outside the window(s) mostly of concrete, buildings, etc. or mostly of natural elements like trees, grass, etc?

(1 = mostly concrete, buildings, etc, 7 = mostly trees, grass, etc)

From the kitchen in your home, is the view outside the window(s) mostly of concrete, buildings, etc. or mostly of natural elements like trees, grass, etc?

(1 = mostly concrete, buildings, etc, 7 = mostly trees, grass, etc)

Does your home have access to a yard with trees, grass, a garden or other natural “green” elements (like bushes)?

(Yes/No)

Does your home have access to a yard with a pond, lake, stream or other natural “blue” elements (like a river)?

(Yes/No)

Please consider your neighborhood/the area where you live. How would you describe the neighborhood/area, in general?

(1 = mostly concrete, buildings, etc, 7 = mostly trees, grass, etc)

How does your neighborhood/area compare to other places?

(1 = far fewer natural “green” elements like trees and grasses, 7 = many more natural “green” elements like trees and grasses)

How does your neighborhood/area compare to other places?

(1 = far fewer natural “blue” elements like lakes and streams, 7 = many more natural “blue” elements like lakes and streams)

There is a park or nature preserve in my local area that is easily accessible.

(1 = strongly disagree, 7 = strongly agree)

There are pleasant natural features in my local area.

(1 = strongly disagree, 7 = strongly agree)

How long would it take to get from your home to the nearest parks or nature reserves?

1 – 5 minutes

6 – 10 minutes

11 – 20 minutes

21 – 30 minutes

30+ minutes

About how much time do you spend outdoors during an average week (please consider all time spent outdoors including time spent working, participating in recreational activities, commuting on foot or via bicycle, etc.)?

Less than an hour

1 – 3 hours

3 – 5 hours

5 – 7 hours

7+ hours

I feel safe being outdoors in the place where I live.

(1 = strongly disagree, 7 = strongly agree)

**Table A. Correlations for Study 1 Variables.**

Measure *1 2 3 4 5 6 7 8 9 10*

1. Nat. Accessibility -- .45*** .22*** .16** .01 .10* -.06 .11** .26*** .24***

2. Nat. From Home .45*** -- .40*** .48*** .10 .14** -.18** .02 .12** .10*

3. Blue Space .22*** .40*** -- .23*** .13 .09 -.01 -.03 .02 -.08

4. GIS Green .16** .48*** .23*** -- .06 .12* -.28***-.01 -.03 .01

5. GIS Blue .01 .10 .13 .06 -- .08 -.04 -.04 -.02 .04

(Immediate)

6. GIS Blue .10* .14** .09 .12* .08 -- .10* -.06 -.01 .05

(Neighborhood)

7. GIS Blue -.06 -.18** -.01 -.28***-.04 .10* -- -.04 .00 -.04

(Broader Locality)

8. AUC .11** .02 -.03 -.01 -.04 -.06 -.04 -- .15*** .20***

9. General .26*** .12** .02 -.03 -.02 -.01 .00 .15*** -- .56***

Health/Wellbeing

10. DASS .24*** .10* -.08 .01 .04 .05 -.04 .20*** .56*** --

*p<.05. **p<.01. ***p<.001. Impulsive Decision-Making measured by AUC (0-1). Higher values indicate less impulsive decision-making, and lower values indicate more impulsive decision-making (see text for details).

**Fig A. Study 1 Direct and Indirect Effects graphs.**


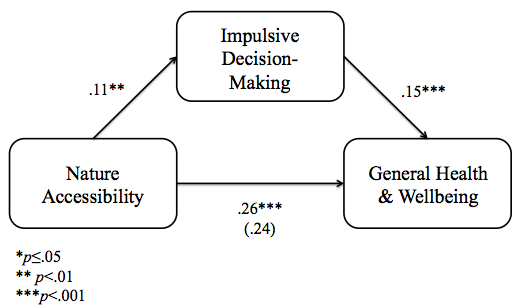


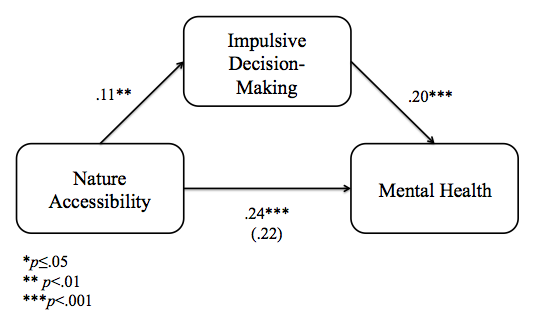


**Table B. Correlations for Study 2 Space Perception Variables.**

Measure *1 2 3*

1. Space Perception #1 -- .30* -.10

2. Space Perception #2 .30* -- .25*

3. Circle Area -.10 .25* --

**p*≤.05

**Fig B. Study 2 Sample Images.**

Nature Condition Built Condition


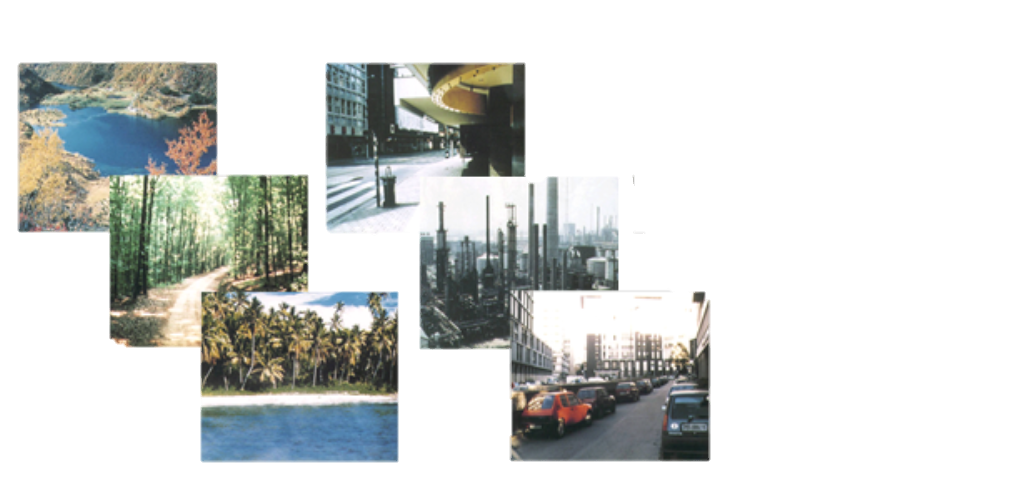


**Fig C. Study 2 Direct and Indirect Effects graph.**


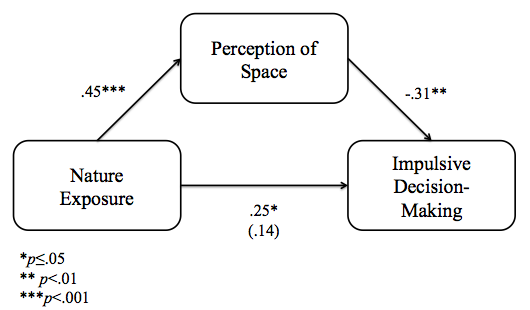

Supplement: S1 Appendix — (DOCX) [file pone.0202246.s001.docx]
